# Fabrication and Characterization of Lignocellulose-Based Porous Materials via Chemical Crosslinking

**DOI:** 10.3390/gels12020140

**Published:** 2026-02-03

**Authors:** Sa Rang Choi, Jung Myoung Lee

**Affiliations:** Department of Wood and Paper Science, Kyungpook National University, 80 Daehakro, Daegu 41566, Republic of Korea; luvvchoi@knu.ac.kr

**Keywords:** lignocellulose, organosolv pulp, microfiber, crosslinking, glycerol diglycidyl ether, aerogels

## Abstract

This study presents a simple method for producing chemically crosslinked porous materials from lignocellulosic fibers with different particle sizes and lignin contents. Porous materials were prepared from organosolv pulp (OP), kneaded organosolv pulp (KOP), lignin-rich microfibrillated cellulose (LMFC), and enzyme cellulose nanofiber (ECNF) and were crosslinked using epichlorohydrin, glutaraldehyde, and glycerol diglycidyl ether (GDE). Among the crosslinkers, GDE provided the best dimensional stability and elastic recovery after repeated compression–recovery cycles in water. Notably, KOP-based porous materials outperformed those derived from LMFC and ECNF, despite being produced via a simple kneading process without energy-intensive fibrillation. KOP-derived materials exhibited excellent dimensional stability and high water absorption exceeding 5890%, demonstrating strong potential for bio-based absorbent applications such as hygiene and packaging.

## 1. Introduction

Absorbent polymers are hydrophilic polymers with a three-dimensional crosslinked structure that can absorb thousands of times their own weight of aqueous solutions [1,2,3,4,5]. However, as the main raw materials for commercial absorbents are petroleum-based polymers, the production of absorbent polymers faces various challenges, including the depletion of petroleum resources, the emission of carbon dioxide, and environmental pollution due to the difficulty of degradation, which has led to the exploration of alternative resources as raw materials.

Cellulose, a representative green biomass resource, is a polymeric material that is abundant in nature. Cellulose fibers can be converted into microfibers such as microfibrillated cellulose (MFC) and cellulose nanofibers (CNFs) through chemical or mechanical processing. Microfibers offer several advantages, including a high specific surface area, chemical modifiability, excellent mechanical properties, and biodegradability. Consequently, numerous studies have focused on preparing functional, eco-friendly polymeric materials, such as aerogels, from microfibers [6,7,8,9,10]. Aerogels, a compound word from “aero” meaning air and “gel” meaning a three-dimensional network structure, are porous nanostructures obtained by replacing the liquid in the gel structure with air. The low density and light weight of aerogels make them versatile platforms for the fabrication of hygroscopic materials, thermal and acoustic insulation, and biological tissue scaffolds.

Typically, porous materials such as aerogels are prepared via supercritical or freeze-drying processes [11,12,13]. However, supercritical CO_2_ drying, which involves a solvent exchange process with ethanol or acetone, requires large initial investments and has high manufacturing costs, making freeze-drying at low temperatures (below −50 °C) and pressures (close to vacuum) more preferable [14]. Porous materials prepared via ice-templating and freeze-drying alone form a three-dimensional pore structure owing to hydrogen bonding between the hydroxyl groups of cellulose fibers [15]. Therefore, the applications of cellulose-based aerogels are limited because these aerogels are easily degraded by contact with water, owing to the breaking of hydrogen bonds upon exposure to water. To overcome this shortcoming, a crosslinking strategy has been proposed, in which a crosslinking agent is added to induce chemical bonding between the fibers [16].

Chemical crosslinking with a crosslinking agent involves adding a crosslinker to a cellulose suspension before drying (for instance, freeze-drying) to produce a porous material with a three-dimensional, netted structure that is resistant to moisture and water. Chemically crosslinked porous materials do not disintegrate when exposed to water, show high dimensional stability, and are highly elastic. Formaldehyde (HCHO)-based crosslinkers are widely used and exhibit excellent crosslinking efficiency. However, formaldehyde is a toxic material that is carcinogenic for humans, necessitating the exploration of “formaldehyde-free” crosslinking agents [17,18,19,20].

CNFs are mainly manufactured via top-down extraction or conversion from high-purity cellulosic raw materials such as bleached chemical pulp. However, these methods have low manufacturing yields and high energy consumption. Therefore, in a previous study, we prepared an organosolv pulp with a shorter fiber length and higher lignin content than commercial bleached kraft pulp and subjected it to alkali kneading to produce micronized/fibrillated fibers [21]. The prepared organosolv pulp had a higher residual lignin content than the kraft pulp, which improved the fabrication yield of micronized/fibrillated fibers compared with that using bleached chemical pulp as the raw material. In addition, kneading machines such as Hobart mixers rotate at relatively low speeds, causing considerable interfiber friction and dispersion, which can lead to energy-efficient micronization of organosolv fibers.

Herein, lignocellulose-based porous materials with different particle sizes and lignin contents were prepared from organosolv pulp obtained through alkali kneading or homogenization of microfibers and MFC, and their properties were compared using CNF as a control material. Porous materials were also prepared using three crosslinkers—epichlorohydrin (ECH), glutaraldehyde (GA), and glycerol diglycidyl ether (GDE) [22]—which have relatively lower toxicity compared with formaldehyde-based crosslinkers. This study aimed to investigate the impact of cellulosic raw material characteristics and crosslinker selection on the microstructure and performance of the resulting porous materials. To achieve this, porous materials with varying lignin content, particle size, and crosslinking chemistries were compared for their structural, water absorption, and mechanical properties.

## 2. Results and Discussion

### 2.1. Fiber Properties

**Figure 1:**
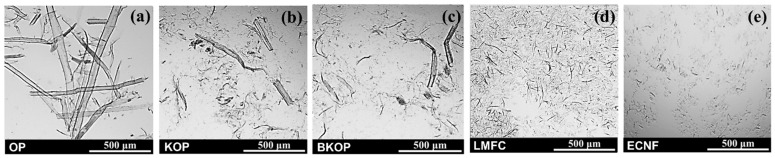
Optical micrographs (×60) of fiber samples: (**a**) OP; (**b**) KOP; (**c**) BKOP; (**d**) LMFC; (**e**) ECNF.

Optical micrographs of the fibers are shown in Figure 1. Alkali-kneaded organosolv pulp (KOP) and bleached and alkali-kneaded organosolv pulp (BKOP), as well as homogenized lignin-rich microfibrillated cellulose (LMFC), show that fibers were modified by mechanical treatments. Compared with the organosolv pulp (OP), the alkali-kneaded samples exhibit smaller fiber sizes, numerous fine particles, and fibrillation. No significant differences in fiber morphology between KOP and BKOP are observed. LMFC also shows a markedly reduced fiber size and an extensive presence of fine fibrillated fragments, indicating that high-pressure homogenization effectively breaks down the fiber bundles into microfibrillar structures. In contrast, Enzyme cellulose nanofiber (ECNF) shows the smallest and most uniformly dispersed fibers, which can be attributed to the combined effects of enzymatic pretreatment [23,24] and a higher number of homogenization passes (six passes for ECNF compared with three passes for LMFC). This intensified treatment further promotes fibrillation, resulting in finer particle sizes than those observed in LMFC [25].

LMFC and ECNF consist predominantly of highly fibrillated fragments. Consequently, these fibrillated components are largely classified as fines by the Kajaani fiber analyzer, which limits the reliability of fiber length and width measurements for these materials. To enable approximate comparisons of fiber lengths across all samples, including LMFC and ECNF, additional fiber length measurements were conducted from the optical micrographs shown in Figure 1, and the results are summarized in Table 1. It should be noted that this image-based analysis is subject to inherent limitations. For OP, long, coarse fibers frequently extended beyond the field of view, resulting in measurements biased toward shorter, fully visible fibers. For KOP and BKOP, fiber length estimation was mainly performed on clearly distinguishable fiber-shaped entities, whereas highly fragmented fines were difficult to identify and thus excluded. Similarly, for LMFC and ECNF, only fiber-like structures were considered, leading to a bias toward relatively larger fibers. Consequently, the reported values represent estimated apparent fiber lengths rather than absolute fiber size distributions. Despite these limitations, the image-based analysis provides useful comparisons of the relative fiber size among the samples. The estimated fiber lengths decrease in the order of OP > KOP ≈ BKOP > LMFC > ECNF. This trend is consistent with the qualitative observations from the optical micrographs. It further supports that alkali kneading produces partially fibrillated fibers with intermediate dimensions, whereas homogenization—particularly when combined with enzymatic pretreatment—leads to extensive fibrillation.

In addition to the image-based fiber length estimation, the dimensional characteristics of OP, KOP, and BKOP were further quantified using the Kajaani fiber analyzer, as summarized in Table 2. Because LMFC and ECNF contain a large proportion of fibrillated fragments smaller than 0.2 mm, most of their particles would be classified as fines in the Kajaani fiber analyzer. As a result, reliable measurements of fiber length and width cannot be obtained for these materials. Therefore, only OP, KOP, and BKOP were evaluated using the Kajaani analyzer. The weighted length of OP is 1.39 mm, the fiber width is 33.93 μm, and the content of fine particles smaller than 0.2 mm is 49.1%. Conversely, the weighted lengths of alkali-kneaded KOP and BKOP are 0.86 and 0.84 mm, respectively, representing decreases of 38.1% and 39.6% compared with OP. Additionally, their fiber widths are 29.26 μm and 28.88 μm, corresponding to decreases of 13.8% and 15.1%, respectively. The contents of fine particles smaller than 0.2 mm increase to 67.6% and 68.3%, which are approximately 18.5% points and 19.1% points higher than that of OP. The observed reductions in weighted fiber length and fiber width, as well as the increased fines content in KOP and BKOP relative to OP, can be attributed to alkali kneading effects on the fiber structure. Ji et al. [26] reported that NaOH treatment induces fiber swelling and peeling reactions. Additionally, combined alkaline and bleaching pre-treatments have been reported to enhance the separation of fiber bundles and promote fibrillation, thereby contributing to fiber individualization and morphological changes that shorten fiber length and increase the proportion of fine particles [27], consistent with the trends observed in this study. Bleaching itself did not significantly alter the overall fiber morphology; however, it resulted in slight decreases in fiber length (6%) and fiber width (1%), accompanied by a minor increase of approximately 1% in the proportion of fines. It can lead to slight reductions in fiber length and width and modest increases in fine particle content due to partial removal of residual lignin and hemicellulose, which facilitates mechanical modification [28]. The coarseness of OP is 0.292 mg/m, whereas those of KOP and BKOP decrease to 0.147 and 0.119 mg/m, corresponding to reductions of 49.7% and 59.2%, respectively, due to alkali kneading. Lower coarseness is generally associated with reduced lignin content, as lignin removal makes the fiber surface smoother and more flexible [29]. Therefore, the lower coarseness values of KOP and BKOP relative to OP are attributed to partial lignin removal during alkali treatment and bleaching.

Table 3 presents the particle-size distributions of KOP, LMFC, and ECNF. The detailed size-distribution profiles are provided in Figure S2. It should be noted that the particle-size analyzer reports an equivalent spherical diameter based on light-scattering principles [30], which may not represent the true dimensions of anisotropic fibers. Nevertheless, the measurements are suitable for comparing the relative degree of micronization among the samples. KOP exhibited a relatively broad particle-size distribution, with 47.3% of particles falling within 0–100 μm and substantial fractions in the 101–300 μm (28.4%) and 301–800 μm (19.8%) ranges. This wide distribution indicates that alkali kneading induces partial micronization but does not achieve extensive fibrillation, leaving a considerable portion of larger fiber fragments. The presence of larger particles (>300 μm) suggests that kneading promotes fiber shortening and partial fibrillation rather than complete disintegration into fine fibrils. These results are consistent with the observations from the optical micrographs of Figure 1. In contrast, LMFC and ECNF showed markedly smaller particle sizes. LMFC contained 87.4% of particles below 100 μm, whereas ECNF, which was produced through enzymatic pretreatment followed by homogenization, exhibited an even more pronounced shift toward ultrafine particles, with 98.9% of particles within the 0–100 μm range and less than 1% exceeding 300 μm. These results confirm that both LMFC and ECNF underwent more intensive fibrillation than KOP, with ECNF showing the highest degree of nanofibrillation among the samples due to the combined enzymatic and mechanical treatments. The extremely low proportions of larger particles in ECNF indicate that enzymatic pretreatment, coupled with homogenization, effectively breaks down the fiber structure to nanoscale dimensions.

Overall, the particle-size comparison demonstrates that alkali kneading produces partially fibrillated fibers with a heterogeneous size distribution, whereas homogenization—especially when combined with enzymatic pretreatment—yields highly uniform micro- to nano-scale fibrils. These differences in fibrillation degree are expected to significantly influence the formation, porosity, and mechanical behavior of the chemically crosslinked porous materials prepared in this study.

The residual lignin contents of all the samples are given in Table 4. The residual lignin content of OP is 24.3%, whereas the alkali-kneaded KOP shows an approximately 6.3 percentage point lower lignin content. Alkali treatment dissolves some of the hemicellulose and lignin from the cell walls, so it was assumed that a significant amount of lignin was removed through alkali kneading [31,32]. BKOP has a lower residual lignin content (3.9%) than KOP because of sodium chlorite bleaching. LMFC, which was micronized via mechanical treatment alone, shows approximately a 2.6 percentage point lower value than OP, presumably because some of the lignin was separated as the fibers were broken by mechanical friction [33]. The ECNF, produced from bleached kraft pulp, contains only 0.1% residual lignin.

### 2.2. Properties of the Prepared Porous Materials

**Figure 2:**
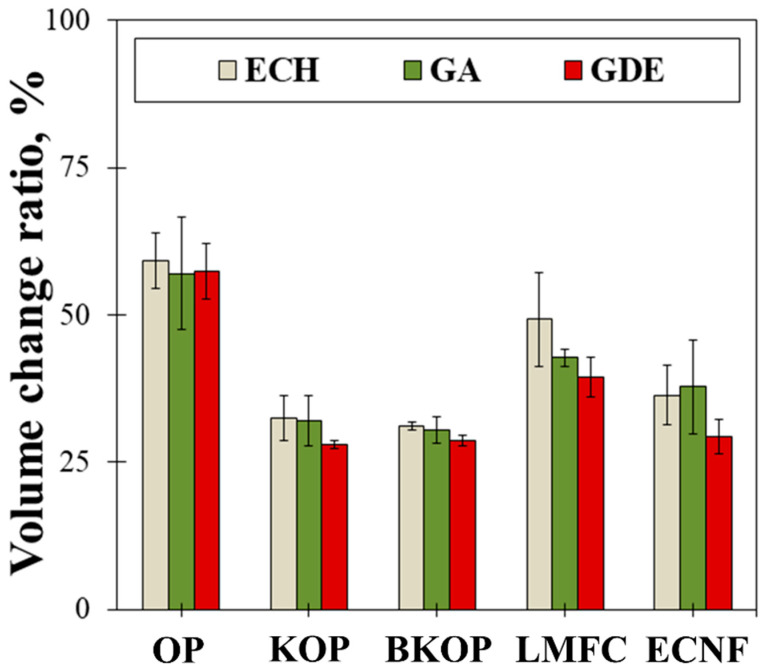
Volume change ratios of the porous materials.

To evaluate how the particle size and lignin content of the raw fibers influence the structural integrity of the resulting porous materials, cellulose-based porous structures were fabricated from five types of lignocellulosic materials (OP, KOP, BKOP, LMFC, and ECNF) using three crosslinkers (ECH, GA, and GDE). Figure 2 shows the measured volume change rates of these porous materials before and after drying. The volume change rates of the porous materials prepared from OP using three crosslinkers (ECH, GA, and GDE) are 59%, 57%, and 58%, respectively. The volume change rates of KOP-based materials are 31%, 31%, and 27%, and those of BKOP-based materials are 30%, 29%, and 28%, respectively. The volume change rates of LMFC-based materials are 49%, 43%, and 40%, respectively, and those of ECNF-based materials are 36%, 38%, and 29%, respectively. The volume change rates of the porous materials prepared from various raw materials decrease in the following order: OP > LMFC > ECNF > KOP > BKOP, with all three crosslinkers showing similar results. This trend can be attributed to the distinct morphological characteristics of the fibers. OP possesses long and coarse fibers that collapse extensively during drying because the hydrogen-bonded network formed during freezing cannot be fully preserved. Conversely, LMFC and ECNF contain highly fibrillated micro- and nanoscale fibers that create a more entangled network, resulting in moderate dimensional stability [34].

KOP and BKOP exhibit the lowest shrinkage, which is likely associated with their partially fibrillated fibers produced through alkali kneading. These fibers may retain a rigid micro-scale framework while providing sufficient surface area for crosslinking. Such a structural combination is expected to contribute to the formation of a mechanically robust three-dimensional network that effectively resists collapse during drying. The slightly improved stability of BKOP over KOP may be related to the removal of residual lignin during bleaching, which increases fiber flexibility and facilitates network formation [35].

Polymers chemically crosslinked with crosslinking agents form a three-dimensional network, and the higher the crosslinking efficiency, the greater the dimensional stability [36]. However, the minimal differences observed among the three crosslinkers indicate that the intrinsic morphology of the raw fibers plays a more dominant role in determining volume stability than the crosslinking chemistry itself. Although the type of crosslinker did not significantly affect the overall dimensional stability of the prepared porous materials, GDE-crosslinked samples showed slightly higher dimensional stability. This tendency may be attributed to the difunctional epoxide structure of GDE, which can promote more uniform and flexible crosslink formation compared with monofunctional epoxy or aldehyde-based [37].

**Figure 3:**
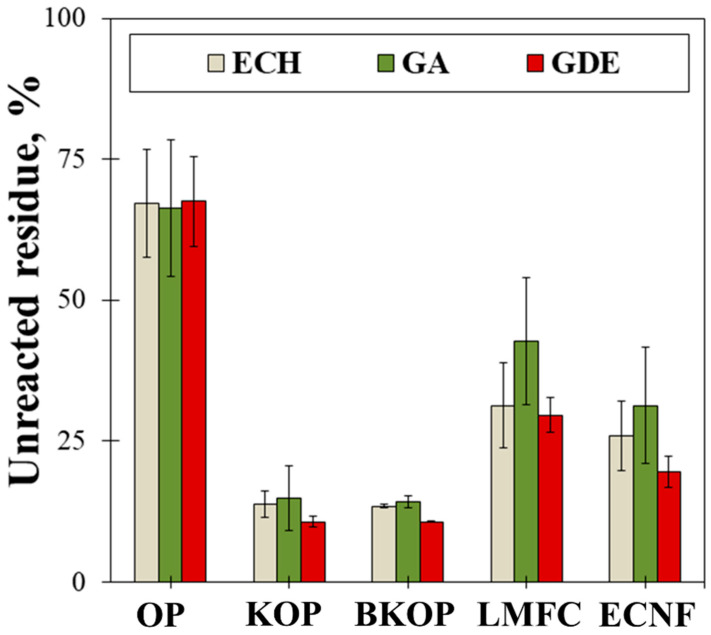
Unreacted residue of the porous materials.

To evaluate the effect of the particle size and lignin content of the raw material and the type of crosslinker on the efficiency of crosslinking between fibers, the content of uncrosslinked fiber residue was measured (Figure 3). In general, in the solid state, polymer chains in polymeric materials exhibit strong intermolecular interactions. However, when polymeric materials are precipitated in a solvent, solvent molecules between the polymer chains cause swelling and gel formation. Eventually, the polymer chains break off completely, dispersing and dissolving in the solvent. However, chemically crosslinked polymers do not dissolve easily in a solvent after swelling; therefore, a low unreacted fiber residue content indicates good crosslinking efficiency. The unreacted OP residues with ECH, GA, and GDE as crosslinkers are 67%, 66%, and 68%, respectively, the highest among the samples. At the same time, KOP-based materials contain 15%, 16%, and 13% unreacted residue, respectively, and BKOP-based materials show the lowest values, 15%, 15%, and 13%, respectively. The unreacted residues of LMFC-based materials with ECH, GA, and GDE as crosslinkers are 31%, 43%, and 30%, and those of ECNF-based materials are 26%, 31%, and 20%, respectively. Therefore, among the three crosslinkers, GDE shows the best reactivity with the fibers. KOP and BKOP exhibit good reactivity with GDE, with BKOP, which has a lower lignin content than KOP, showing slightly better or similar crosslinking efficiency. For LMFC and ECNF, which consist of very fine particles, a greater number of crosslinking agents may be required due to their high specific surface area and high number of reactive sites.

**Figure 4:**
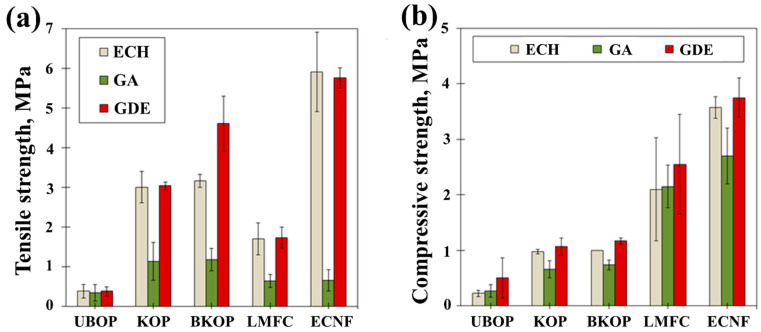
Mechanical properties of the porous materials: (**a**) tensile strength; (**b**) compressive strength.

Figure 4 shows the tensile and compressive strengths of the porous materials prepared from different lignocellulosic fibers and crosslinkers. Among the raw materials, ECNF-based porous materials consistently exhibited the highest tensile and compressive strengths, followed by LMFC, BKOP, KOP, and OP. This trend can be attributed to the progressive reduction in fiber size and the corresponding increase in specific surface area from OP to ECNF. The high surface area and abundant hydroxyl groups of ECNF promote extensive inter-fiber hydrogen bonding, resulting in a dense and mechanically robust network. KOP- and BKOP-based materials showed intermediate strength values, indicating that alkali kneading effectively enhances mechanical performance by inducing partial fibrillation while preserving a rigid micro-scale framework. The slightly higher strength of BKOP compared with KOP is likely related to lignin removal during bleaching, which improves fiber flexibility and facilitates more efficient network formation. In contrast, OP-based materials exhibited the lowest strength due to their coarse fiber morphology and limited inter-fiber bonding.

Regarding the type of crosslinker, GDE-crosslinked materials exhibited the highest compressive strength, followed by ECH- and GA-crosslinked materials, regardless of fiber type. Compared with epoxide-based crosslinkers that react directly with the abundant hydroxyl groups of cellulose to form stable ether linkages under alkaline conditions, aldehyde-based crosslinkers such as glutaraldehyde predominantly react with amine groups and form hemiacetal/acetal structures in the presence of catalysts or specific conditions [38]. These results indicate that variations in fiber morphology, surface chemistry, and crosslinking chemistry collectively affect the mechanical performance of the porous materials, with epoxy-based crosslinkers exhibiting better performance than the other examined crosslinkers.

**Figure 5:**
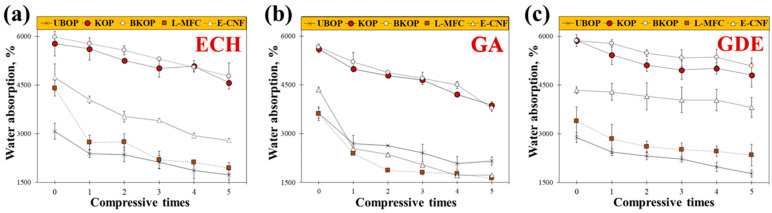
Effect of crosslinking agent on the water absorption rates of the prepared porous materials; (**a**) ECH; (**b**) GA; (**c**) GDE.

Figure 5a presents the changes in water absorption capacity of the porous materials crosslinked with ECH during repeated compression–recovery cycles. OP exhibited an initial water absorption of 3078%, which decreased to 1728% after five cycles. In contrast, KOP and BKOP showed much higher initial absorption values of 5298% and 5981%, respectively, and retained relatively high absorption capacities of 4790% and 4951% after five cycles. LMFC and ECNF exhibited initial absorption values of 4405% and 4737%, which decreased to 1941% and 3196%, respectively, after repeated compression. Figure 5b shows a similar trend for GA-crosslinked porous materials. KOP- and BKOP-based samples consistently exhibited higher initial water absorption and better retention after repeated compression compared with OP, LMFC, and ECNF. However, the overall absorption retention of GA-crosslinked materials was lower than that of ECH-crosslinked counterparts, indicating less effective structural recovery under mechanical deformation. These results demonstrate that the water absorption behavior of the porous materials under repeated compression–recovery cycles is strongly influenced by both the characteristics of the lignocellulosic fibers and the type of crosslinking agent. Across all crosslinkers, porous materials derived from KOP and BKOP consistently exhibited higher initial water absorption capacities and superior retention after repeated compression than OP-, LMFC-, and ECNF-based materials.

OP-based porous materials showed the lowest initial absorption and the most pronounced reduction after compression cycling, which can be attributed to their coarse fiber structure and limited network integrity. Conversely, KOP and BKOP-based porous materials maintained high water absorption capacities even after five compression cycles, regardless of the crosslinker used. Although LMFC and ECNF consist of highly fibrillated micro- and nanoscale fibers with high specific surface areas, their water absorption capacities decreased more substantially after repeated compression. This suggests that excessive fibrillation may lead to a denser, more compact network that is prone to irreversible pore collapse under mechanical stress, thereby limiting water uptake recovery [39]. Among the crosslinkers, GDE-crosslinked porous materials exhibited the highest water retention during compression cycling. This behavior can be attributed to the difunctional epoxide structure of GDE, which enables more flexible, uniformly distributed crosslinking within the fiber network. In contrast, GA-crosslinked materials generally showed greater reductions in water absorption, likely due to the formation of more rigid, heterogeneous crosslinking domains that are less effective at accommodating repeated deformation.

These findings indicate that achieving an optimal balance between partial fiber fibrillation and flexible chemical crosslinking is crucial for creating porous materials with high water absorption capacity and mechanical durability under repeated compression. In this regard, alkali-kneaded fibers combined with epoxy-based crosslinkers, particularly GDE, appear to offer a favorable structural configuration for absorbent applications that require repeated mechanical loading. Additionally, compared to aldehyde-based crosslinkers, GDE has been reported to exhibit improved biostability and reduced cytotoxicity, making it a more suitable candidate for crosslinking cellulose-based porous materials in applications where biocompatibility is a consideration [22,37].

**Figure 6:**
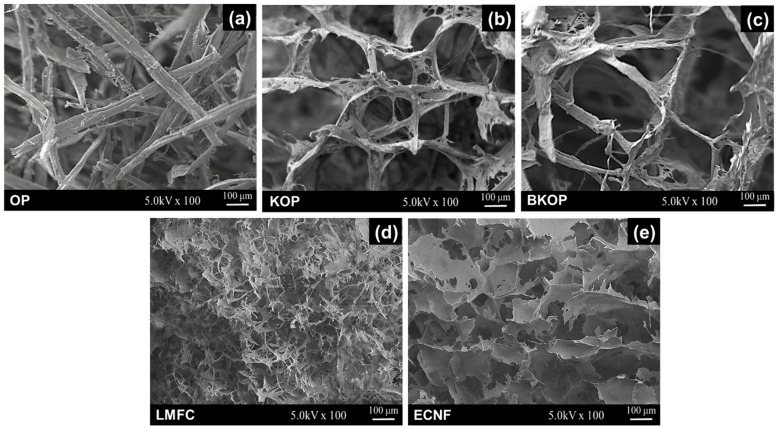
SEM images of porous structures prepared from (ligno) cellulose cross-linked via GDE; (**a**) OP; (**b**) KOP; (**c**) BKOP; (**d**) LMFC; (**e**) ECNF.

Based on the superior dimensional stability, mechanical performance, and water absorption retention observed for GDE-crosslinked porous materials, their microstructures were further examined by SEM to elucidate the underlying structural features. Figure 6 shows the SEM images of porous materials prepared from OP, KOP, BKOP, LMFC, and ECNF using GDE as the crosslinking agent. The OP-based porous material (Figure 6a) exhibits a relatively coarse, loosely connected fiber network, characterized by large, irregular pores. Such a structure is consistent with the pronounced volume shrinkage and limited mechanical robustness observed for OP-based samples, as the weakly interconnected framework is susceptible to collapse during drying and mechanical deformation.

In contrast, the KOP- and BKOP-based porous material (Figure 6b,c) shows a well-developed three-dimensional network with more uniform pore sizes and thicker pore walls. The partially fibrillated fibers form an interconnected microscale framework that appears mechanically robust while maintaining sufficient open porosity. This microstructure is in good agreement with the excellent dimensional stability and high water absorption retention of KOP- and BKOP-based materials under repeated compression. Compared with LMFC, the ECNF-based porous material shows a more compact, denser structure (Figure 6d,e). This difference is likely related to enhanced hydrogen bonding between the nanoscale fibrils in ECNF, arising from their smaller dimensions, higher specific surface area, lower residual lignin content, and abundant hydroxyl groups, which collectively facilitate closer inter-fibrillar interactions and lead to a more compact network structure.

To examine the influence of fiber size and fibrillation degree on the specific surface area of the porous materials, BET (Brunauer–Emmett–Teller) surface area analysis was performed for OP-, KOP-, and ECNF-based samples (Table 5). These materials represent coarse fibers, partially fibrillated fibers obtained by alkali kneading, and highly fibrillated commercial cellulose nanofibers, respectively. The OP-based porous material exhibited the lowest specific surface area (4.5 m^2^/g). In contrast, the KOP-based porous material showed a markedly higher surface area of 7.2 m^2^/g, approximately 60% higher than that of OP. Alkali kneading induces partial fibrillation, resulting in finer fibers and a more interconnected porous network, which increases pore wall area and surface accessibility, as observed in the SEM images. The ECNF-based porous material exhibited the highest surface area (8.4 m^2^/g). However, the difference in specific surface area between KOP and ECNF was relatively small (approximately 16%), indicating that partial fibrillation achieved by alkali kneading is sufficient to generate a highly accessible porous structure.

**Figure 7:**
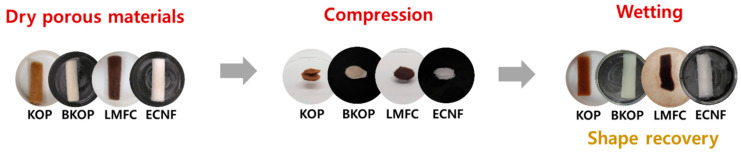
Images of the porous materials recovering shape in water.

Consequently, porous materials prepared from KOP and BKOP exhibited the highest water absorption capacities and the most effective shape recovery under repeated compression–recovery cycles, as shown in Figure 7. Their rapid and reversible structural recovery upon rewetting is further illustrated in Supporting Information Video S1. These findings demonstrate that high-performance lignocellulose-based porous materials can be achieved without relying on energy-intensive fibrillation processes. Instead, an appropriate balance between partial fiber fibrillation and flexible chemical crosslinking enables the formation of porous structures that combine high absorbency, mechanical robustness, and recoverability under repeated deformation. Accordingly, such porous materials are expected to be promising candidates for applications requiring repeated wetting, compression, and recovery, such as moisture-control materials, hygienic absorbents, cushioning packaging, and bio-related absorbent systems.

## 3. Conclusions

In this study, chemically crosslinked porous materials were successfully prepared from lignocellulosic materials with different particle sizes and lignin contents using various crosslinking agents. The volume change rates decreased in the order of OP > LMFC > ECNF > KOP > BKOP, demonstrating that partially fibrillated fibers obtained by alkali kneading provide superior dimensional stability compared with both coarse fibers and excessively fibrillated micro- or nanofibers. Among the crosslinkers examined, glycerol diglycidyl ether (GDE) generally produced porous materials with the best dimensional stability. In contrast, glutaraldehyde (GA) resulted in inferior mechanical properties. Porous materials derived from KOP and BKOP exhibited exceptionally high water absorption capacities exceeding 5890% and maintained their structural integrity under repeated compression–recovery cycles. All chemically crosslinked porous materials remained insoluble in water and demonstrated rapid shape recovery upon rewetting, retaining their functionality over at least five compression cycles. These findings underscore the importance of balancing partial fiber fibrillation with flexible chemical crosslinking to achieve porous materials with high absorbency, mechanical robustness, and a recoverable structure. In particular, alkali-kneaded fibers combined with GDE crosslinking emerge as a promising strategy for developing lignocellulose-based porous materials suitable for applications requiring repeated wetting, compression, and recovery, such as hygienic absorbents, packaging, and bio-related absorbent systems.

## 4. Materials and Methods

### 4.1. Materials

#### 4.1.1. Organosolv Pulp (OP)

Organosolv pulp was fabricated from Chilean radiata pine chips, with a moisture content of 5.7 ± 0.3% and an alkali-extractable component content of 6.3 ± 0.4%. The chips were 35 (±3) mm high, 8 (±3) mm wide, and 7 (±2) mm thick. The reactive solvent used for organosolv pulping was glycol ether (Pure grade, Sigma-Aldrich Co., St. Louis, MO, USA), and sulfuric acid (95% purity, Daejung Chemicals & Metals, Siheung, Gyeonggi-do, Republic of Korea) was used as a catalyst. Wood chips and a glycol ether–sulfuric acid mixture (glycol ether and sulfuric ratio of 97:3, *v*/*v*) were mixed at a solid-and-liquid ratio of 1:2 (*w*/*v*). The mixture was treated in a high-pressure steam unit (autoclave, HST 506-6, Hanbaek ST, Guangju, Republic of Korea) at 120 °C for 120 min. Subsequently, the reaction mixture was filtered and washed with distilled water to remove lignin and distillate. The yield of the prepared organosolv pulp was approximately 60%.

#### 4.1.2. Kneaded Organosolv Pulp (KOP)

OP was subjected to alkali kneading to determine the effect of fiber particle size on the properties of crosslinked porous materials. A 0.25N sodium hydroxide (NaOH, 97% purity, Daejung Chemicals & Metals, Siheung, Gyeonggi-do, Republic of Korea) solution was used to dilute OP to 10%. A 1000 mL aliquot of diluted pulp slurry was injected into a laboratory Hobart Mixer (N50, Hobart Corporation, Troy, OH, USA), which has a maximum capacity of 4500 mL, and treated for 3 h at room temperature at 281 rpm. After kneading, it was filtered with 0.5N NaOH to separate lignin and washed with water until neutral. The fabrication yield of the kneaded microfibers was 90.6 ± 2.1%.

#### 4.1.3. Bleached and Kneaded Organosolv Pulp (BKOP)

To control the residual lignin content of the microfibers prepared via alkali kneading, the microfibers were delignified using sodium chlorite. After diluting 10 g of KOP to a concentration of 3%, 1 g of sodium chlorite (78% purity, Daejung Chemicals & Metals, Siheung, Gyeonggi-do, Republic of Korea) and 0.2 mL of acetic acid (99% purity, extra pure grade, Duksan, Jincheon, Republic of Korea) were added. Sodium chlorite (1 g) and acetic acid (0.2 mL) were added three times at 1 h intervals, with stirring, in a constant-temperature water bath at 70 °C. At the end of the reaction, the mixture was washed with distilled water under reduced pressure until the filtrate was neutral.

#### 4.1.4. Lignin-Rich Microfibrillated Cellulose (LMFC)

A high-pressure homogenizer (Panda Plus 2000, GEA Niro Soavi., Parma, Italy) was used to prepare LMFC. OP was diluted to 1.5% and processed three times at 1000 bar. The solid content of the prepared LMFC was 1.3%.

#### 4.1.5. Enzyme Cellulose Nanofiber (ECNF)

Commercial ECNF with a 2% solid content was provided by a Moorim P&P Co., Ltd. (Ulsan, Republic of Korea). According to the manufacturer, the ECNF was produced from hardwood bleached kraft pulp pretreated with endo-glucanase and subsequently processed through six cycles of high-pressure homogenization (Ariete NS311OH, GEA Niro Soavi, Parma, Italy) at 800–1000 bar.

#### 4.1.6. Crosslinking Agent and Catalyst

The crosslinking agents used in this study were ECH (Guaranteed Reagent, Junsei Chemical Co., Ltd., Chuo-ku, Tokyo, Japan), GA (Chemical pure, Daejung Chemical & Metals Co., Ltd., Siheung, Gyeonggi-do, Republic of Korea), and GDE (Technical grade, Sigma-Aldrich Co., Ltd., Louis, MO, USA). NaOH (Guaranteed Reagent, Daejung Chemicals & Metals Co., Ltd., Siheung, Gyeonggi-do, Republic of Korea) and aluminum sulfate (Extra pure, Daejung Chemical & Metals Co., Ltd., Siheung, Gyeonggi-do, Republic of Korea) were used as catalysts.

### 4.2. Fabrication of Porous Material

Lignocellulose-based porous materials were prepared from lignocellulosic materials produced in various ways (Figure S1). The pulp was diluted to 1% with distilled water, and the crosslinker and catalyst were added at a 1:1:1 molar ratio. Three crosslinkers were used: ECH, GA, and GDE. When using ECH and GDE as crosslinkers, NaOH was employed as a catalyst, and with GA as the crosslinker, aluminum sulfate was used as the catalyst. After stirring for approximately 5 min using a stirrer, the reaction was conducted in a constant-temperature water bath at 60 °C for 1 h. After the reaction, the pulp slurry was placed in a cylindrical tube with a diameter of 14 mm and a height of 40 mm and frozen for 6 h at −28 °C. It was then freeze-dried (TFD5505A, Dongducheonsi, Gyeonggido, Republic of Korea) for 48 h.

### 4.3. Measurement

#### 4.3.1. Fiber Characterization

The morphology of the fibers was observed using optical microscopy (BX 50, Olympus Optical Co. Ltd., Hachioji, Tokyo, Japan) at a magnification of 60× to determine the morphological characteristics of the fibers.

Fiber apparent lengths were measured from optical micrographs using ImageJ software 1.48v (National Institutes of Health, Bethesda, MD, USA). Only fibers that could be clearly distinguished as individual fiber-like entities were randomly selected (*n* ≥ 30), whereas highly entangled fibers or indistinguishable fine fibrils were excluded from the analysis.

The average fiber length, average fiber width, content of fine particles (<0.2 mm), and fiber coarseness were measured using a fiber analyzer (Kajaani FS300, Metso Automation, Helsinki, Finland), and the particle sizes of KOP, LMFC, and ECNF, which were micronized via homogenization, were determined using a particle size analyzer (Mastersizer 3000, Malvern Instruments Ltd., Worcestershire, UK). The acid-insoluble lignin content was analyzed according to the TAPPI T 222 om-15 method. To a sample of 0.2 g total dry weight, 5 mL of 72% sulfuric acid was added, and the reaction was conducted at room temperature for approximately 4 h. At the end of the reaction, to induce hydrolysis, 196 mL of distilled water was added, and the mixture was treated at 120 °C for 120 min in a high-pressure steam autoclave (HST 506-6, Hanbaek ST, Guangju, Republic of Korea). After the end of hydrolysis, the filtrate was washed with distilled water until neutral and dried in a desiccator at 105 °C ± 3 °C for 12 h.

#### 4.3.2. Characterization of the Porous Materials

To assess the dimensional stability of the fabricated porous materials, the volume change rates before and after drying were calculated using Equation (1):

(1) $$Volume\;change\;rate\;(\%)\;=\;\frac{V_{2}-\;V_{1}}{V_{1}}\times 100$$

where *V*_1_ denotes the volume of porous foam before freeze-drying, and *V*_2_ represents the volume of porous foam after freeze-drying.

Additionally, the unbound, nonreactive residue content was measured to determine the efficiency of crosslinking between lignocellulose and the crosslinking agent. Approximately 0.5 g of the dry porous material was placed in a 50 mL conical tube, and approximately 40 mL of distilled water was added. Then, the mixture was stirred for 5 min at level 7 using a vortex mixer (Vortex-Genie, Scientific Industries, Bohemia, NY, USA). The unreacted residue dispersed in distilled water was filtered under vacuum through filter paper (Advantec No. 2, Tokyo, Japan) and dried in a desiccator at 105 °C for 12 h. The content of unreacted residue was calculated using Equation (2):

(2) $$Nonreactive\;residue\;content\;(\%)\;=\;\frac{w}{w_{0}}\times 100$$

where *W* denotes the weight of residue after vortex mixing (g), and *W*_0_ represents the weight of the sample before vortex mixing (g).

A tensile strength tester (Hounsfield H500M, Redhill, Surrey, UK) was used to measure the tensile and compressive strengths of the porous materials. For the tensile test, specimens with a height of 20 mm and a diameter of 10 mm were used, with a load of 490 N on the load cell and a tensile speed of 2 mm/min. In the compression test, specimens with a height of 10 mm and a diameter of 10 mm were loaded at 1 mm/min, with the ultimate strain set to 75%. The average tensile and compressive strengths were calculated from three measurements for each sample.

The cross-sectional morphology of the porous materials was examined at 100× magnification using a scanning electron microscope (SEM, JSM-7900F, JEOL Ltd., Mitaka, Tokyo, Japan) to characterize the internal pore structure. The sample was removed from the water and allowed to drain by hanging for 5 min to remove excess surface water, and the water absorption rate was calculated using Equation (3):

(3) $$Water\;absroption\;rate\;(\%)=\;\frac{\left(W_{t}-W_{0}\right)}{W_{0}}$$

where *W_t_* denotes the weight of porous material after 24 h of water immersion (g), and *W*_0_ represents the Dry weight of the porous material (g).

To determine the elasticity and recoverability of water-immersed porous materials, they were repeatedly compressed using a tensile strength tester (Hounsfield H500M, Redhill, Surrey, UK) at a strain of 95%, a compression force of 490 N, and a strain rate of 1 mm/min. After the compressed porous material was re-immersed in distilled water for 24 h, the water absorption rate and thickness were measured in the same way to determine the rate of change before and after compression. The recovery test was repeated five times, and the mean and standard deviation were calculated.

The specific surface area was analyzed using a surface area and pore analyzer (NOVA 2000, Quantachrome Instruments, Boynton Beach, FL, USA). The measurable specific surface area range of the BET instrument is from 0.01 to over 2000 m^2^/g.

## Figures and Tables

**Table 1:** Estimated fiber lengths determined by optical image analysis (*n* ≥ 30).

Sample	Fiber Length (μm)	
	Mean	Standard Deviation
OP	0.752	0.308
KOP	0.159	0.120
BKOP	0.153	0.114
LMFC	0.062	0.036
ECNF	0.023	0.010

**Table 2:** Dimensional characteristics of the obtained organosolv pulp fibers and alkali-kneaded fibers.

Sample	Arithmetic Length (mm)	Weighted Length (mm) *	Fiber Width (μm)	Fine Content (%)	Coarseness (mg/m)
OP	0.43	1.39	33.93	49.1	0.292
KOP	0.24	0.86	29.26	67.6	0.147
BKOP	0.23	0.84	28.88	68.2	0.120

* average weighted fiber length.

**Table 3:** Particle-size distributions of KOP, LMFC, and ECNF measured by laser diffraction.

Particle Size (μm)	Sample		
	KOP	LMFC	ECNF
0–100	47.3	87.4	98.9
101–300	28.4	10.0	0.4
301–800	19.8	2.3	0.5
801–1050	4.4	0.3	0.2

**Table 4:** Residual lignin contents of the samples.

Sample	Klason Lignin (%)
OP	24.3 ± 0.4
KOP	18.0 ± 0.1
BKOP	3.9 ± 0.4
LMFC	21.7 ± 0.2
ECNF	0.1 ± 0.0

**Table 5:** BET specific surface areas of OP-, KOP-, and ECNF-based porous materials.

Sample	OP	KOP	ECNF
Surface area (m^2^/g)	4.5	7.2	8.4

## Data Availability

The original contributions presented in this study are included in the article. Further inquiries can be directed to the corresponding author.

## Supplementary Materials

The following supporting information can be downloaded at: https://www.mdpi.com/article/10.3390/gels12020140/s1, Figure S1. Schematic illustration of the preparation process for various pulp-derived materials, including UBOP, KOP, BKOP, L-MFC, and E-CNF. Figure S2. Particle size distribution of UBKOP, L-MFC, and E-CNF. Video S1: Video of post-compressed water swelling behavior of KOP/GDE-based porous materials.

## Abbreviations

The following abbreviations are used in this manuscript:

BET	Brunauer–Emmett–Teller
BKOP	Bleached and alkali-kneaded organosolv pulp
CNF	Cellulose nanofiber
CO_2_	Carbon dioxide
ECH	Epichlorohydrin
ECNF	Enzyme cellulose nanofiber
GA	Glutaraldehyde
GDE	Glycerol diglycidyl ether
KOP	Alkali-kneaded organosolv pulp
LMFC	Lignin-rich microfibrillated cellulose
MFC	Microfibrillated cellulose
NaOH	Sodium hydroxide
OP	Organosolv pulp
SEM	Scanning electron microscopy
