# Supplementary material for: Fabrication and Characterization of Lignocellulose-Based Porous Materials via Chemical Crosslinking
Source: Gels. 2026 Feb 3;12(2):140. doi: 10.3390/gels12020140 (PMC12940017; doi:10.3390/gels12020140)
Supplement: Supplementary file 1 [file gels-12-00140-s001.zip › Supplementary Files/Supplymentary Materials.docx]

**Supplementary Materials**

Fabrication and characterization of lignocellulose-based porous materials via chemical crosslinking

Sa Rang Choi^1^ and Jung Myoung Lee^2*^

^1^ Department of Wood and Paper Science, Kyungpook National University, 80 Daehakro, Daegu, 41566, Republic of Korea; luvvchoi@knu.ac.kr

^2^ Department of Wood and Paper Science, Kyungpook National University, 80 Daehakro, Daegu, 41566, Republic of Korea; jmylee@knu.ac.kr

***** Correspondence: jmylee@knu.ac.kr; Tel.:+82539505796


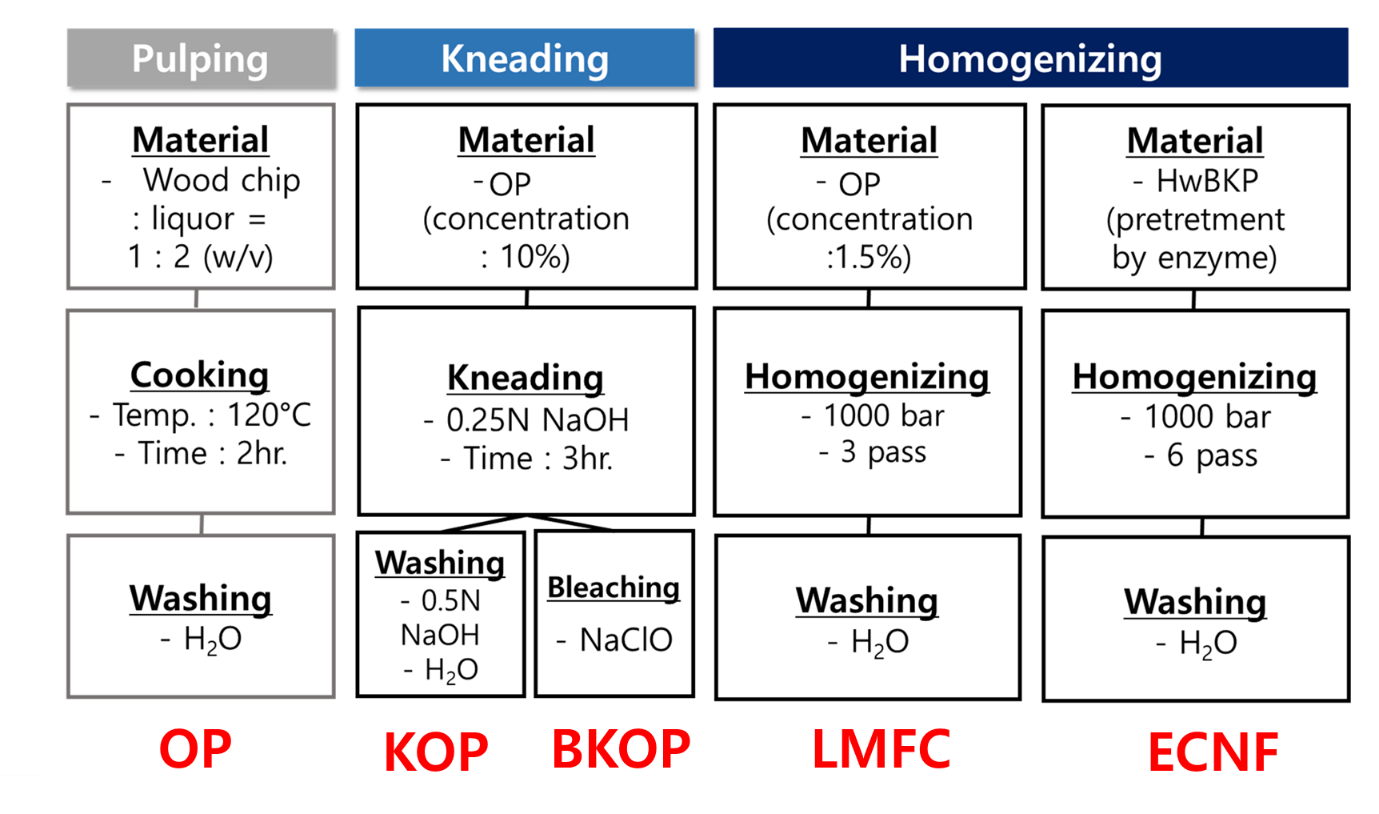


**Figure S1.** Schematic illustration of the preparation process for various pulp-derived materials, including UBOP, KOP, BKOP, L-MFC, and E-CNF.


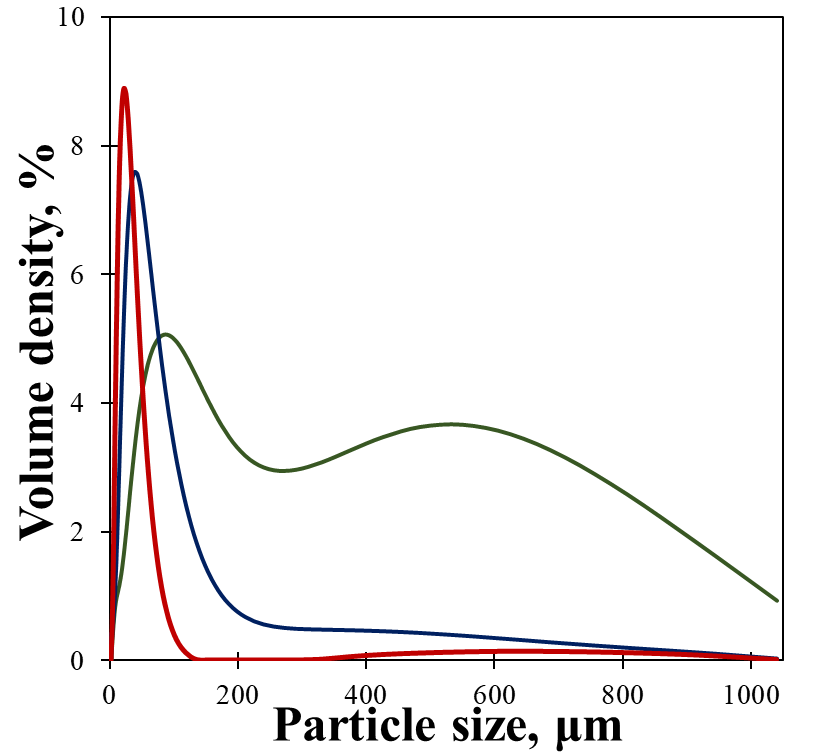


**Figure S2.** Particle size distribution of UBKOP, L-MFC, and E-CNF.
